# Genomic Analysis and Stability Evaluation of the Phenol-Degrading Bacterium *Acinetobacter* sp. DW-1 During Water Treatment

**DOI:** 10.3389/fmicb.2021.687511

**Published:** 2021-07-13

**Authors:** Qihui Gu, Moutong Chen, Jumei Zhang, Weipeng Guo, Huiqing Wu, Ming Sun, Lei Wei, Juan Wang, Xianhu Wei, Youxiong Zhang, Qinghua Ye, Liang Xue, Rui Pang, Yu Ding, Qingping Wu

**Affiliations:** ^1^Guangdong Provincial Key Laboratory of Microbial Safety and Health, State Key Laboratory of Applied Microbiology Southern China, Guangdong Institute of Microbiology, Guangdong Academy of Sciences, Guangzhou, China; ^2^College of Food Science, South China Agricultural University, Guangzhou, China; ^3^Department of Food Science & Technology, Institute of Food Safety and Nutrition, Jinan University, Guangzhou, China

**Keywords:** genome, phenol-degrading, microbial immobilization, seeded bacteria, biofilter

## Abstract

Phenol is a toxic organic molecule that is widely detected in the natural environment, even in drinking water sources. Biological methods were considered to be a good tool for phenol removal, especially microbial immobilized technology. However, research on the “seed” bacteria along with microbial community analysis in oligotrophic environment such as drinking water system has not been addressed. In this study, *Acinetobacter* sp. DW-1 with high phenol degradation ability had been isolated from a drinking water biofilter was used as seeded bacteria to treat phenol micro-polluted drinking water source. Meanwhile, the whole genome of strain DW-1 was sequenced using nanopore technology. The genomic analysis suggests that *Acinetobacter* sp. DW-1 could utilize phenol via the β-ketoadipate pathway, including the catechol and protocatechuate branches. Subsequently, a bio-enhanced polyhedral hollow polypropylene sphere (BEPHPS) filter was constructed to investigate the stability of the seeded bacteria during the water treatment process. The denatured gradient gel electrophoresis (DGGE) profile and the quantification of phenol hydroxylase gene results indicate that when the BEPHPS filter was operated for 56 days, *Acinetobacter* sp. was still a persistent and competitive bacterium in the treatment group. In addition, 16S rRNA gene amplicon sequencing results indicate that *Acinetobacter* sp., as well as *Pseudomonas sp.*, *Nitrospira* sp., *Rubrivivax* sp. were the predominant bacteria in the treatment group, which were different from that in the CK group. This study provides a better understanding of the mechanisms of phenol degradation by *Acinetobacter* sp. DW-1 at the gene level, and provides new insights into the stability of seeded bacteria and its effects on microbial ecology during drinking water treatment.

## Introduction

Phenol is a crucial raw organic material that is widely used in industrial production, pharmaceutical manufacturing, and petroleum refining (Liu et al., 2020). However, the extensive use and discharge of phenol have major impacts on the ecological environment, as phenol is generally toxic to living organisms (Ma Y. et al., 2020). Many physicochemical treatment techniques have been adopted to deal with phenol-contaminated water, including reverse osmosis, electrochemical oxidation, nanofiltration, ultraviolet radiation, ion exchange, adsorption, and immobilized enzymes. However, these methods are expensive, including operation and maintenance costs, require large energy inputs, and produce secondary pollution (Gu et al., 2016). Biological treatment technology has great potential and competitive advantages related to environmental safety and friendliness, as it produces no secondary pollution and has a lower cost (Polivtseva et al., 2020). Biological biodegradation has been widely used for removing phenol from a wide range of environments, including soil (Sharma and Lin, 2017; Gong et al., 2021), wastewater (Liu et al., 2016; Barik et al., 2021), and aquaculture system (Nandi et al., 2020). In addition, these phenol-degrading strains have been successfully used for the bioremediation of the contaminated environments. Nevertheless, phenol-degrading microorganisms isolated from drinking water system were still very limited. Bioremediation of phenol in drinking water system was even scarcer. To our knowledge, drinking water system is oligotrophic environment, isolation of indigenous contaminant-degrading bacteria was very important in this environment. The reason is that indigenous contaminant-degrading bacteria would not bring in alien species that may change the microbial ecology in drinking water systems. In addition, the indigenous bacteria always exhibit a good adaptability to this oligotrophic environment. According to our previous work (Gu et al., 2016), a strain (*Acinetobacter* sp. DW-1) with excellent ability of phenol degradation at both low and high concentration was obtained from drinking water biofilter. Meanwhile, strain DW-1 that immobilized on sterilized biological activated carbon (BAC) and polyhedral hollow polypropylene sphere (PHPS) also exhibited a good ability to remove phenol, among which PHPS are often used as an immobilization material in the pre-treatment of drinking water sources in China. Therefore, we assume that *Acinetobacter* sp. DW-1 may have a great potential of being used as bioremediation in phenol-polluted drinking water source. However, the stability and ecological effects of this strain on microbial community during water treatment are unknown. In addition, to our best knowledge, *Acinetobacter* spp. could degrade phenol via meta- (Ahmad et al., 2017) or ortho-pathways (Lin, 2017). Bacteria in the *Acinetobacter* spp. play an important role in mineralization of aromatic compounds (Wang W. et al., 2019). The genome mining of bacteria will provide insights about the genetic information and functional properties of various proteins and enzymes involved in the microbial metabolism (Ganesh Kumar et al., 2021). To the best of our knowledge, only limited reports are available on complete genome studies using three-generation sequencing technology on phenol degrading bacteria. In order to better understand of phenol degradation mechanisms of *Acinetobacter* sp. DW-1 and then modify for engineering bacteria, genome based on three-generation sequencing technology was needed. Furthermore, more genetic information of *Acinetobacter* sp. DW-1 on the degradation of other pollutants could be obtained. In this study, in order to achieve its further and better application in phenol-polluted drinking water source, the strain DW-1 could be used as seed bacteria in the treatment of biological pretreatment process in view of existing biological treatment processes in drinking water treatment systems, which mainly includes biological pre-treatment and biofiltration processes. However, in biological treatment technology, especially microbial immobilization technology has greatly improved the processing efficiency (Liu et al., 2009; Bramhachari et al., 2016), can resist changes in the external environment, and has a greater application potential (Shin et al., 2019). To our knowledge, the stability of the microorganisms in biological treatment systems has a significant influence on the effectiveness of the treatment. In particular, the stability of core functional microorganisms in the treatment system has great practical significance for evaluating the application value of functional bacteria. Currently, research regarding biological treatment technology such as the application of artificially screened functional bacteria in drinking water treatment is still lacking. Furthermore, research regarding the stability of indigenous specific contaminant-degrading bacteria used as seeded bacteria for drinking water treatment was very limited. What’s more, research on seeded bacteria along with microbial community analysis during water treatment is also urgently needed. Gao et al. (2010) isolated organic micropollutant-degrading bacteria from drinking water sources and immobilized them on biologically activated carbon. However, these functional bacteria were not able to degrade specific pollutants. Davidson et al. (2011) screened bromate-reducing bacteria and immobilized them on activated carbon to treat drinking water, but did not track the changes in the bromate-reducing bacteria during the water treatment process. Wang et al. (2015) isolated a nitrosamine-reducing bacterium from biological activated carbon (BAC) filters, but did not mention its application for actual water bodies. Therefore, additional research is required to evaluate the stability of functional bacteria in practical water applications, and to determine whether these fortified bacteria are still present as dominant bacteria after long-term water treatment. Furthermore, The effect of seeded bacteria on microbial community in water treatment system was also need to be evaluated.

**FIGURE 1:**
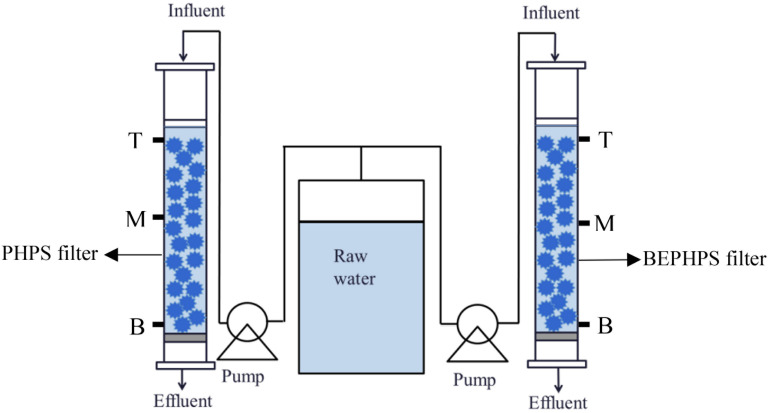
Schematic diagram of the BEPHPS filter used for the water treatment experiments.

In this study, genomic sequencing of *Acinetobacter* sp. DW-1 was performed using third-generation sequencing technology. Subsequently, *Acinetobacter* sp. DW-1 was immobilized on polyhedral hollow polypropylene spheres (PHPS), and then a bio-enhanced PHPS (BEPHPS) filter was constructed using the *A*cinetobacter sp. DW-1-immobilized PHPS, as shown in Figure 1 and Supplementary Figure 1. In addition, the bacterial community compositions and microbial activity in the PHPS biofilms were examined during 56 days of BEPHPS filter operation. The objectives of this study were to explored the phenol degradation mechanism of *Acinetobacter* sp. DW-1, and evaluate the stability of seeded bacteria during the water treatment process, as well as microbial interactions in the process of bioremediation, and explore the application potential of strain DW-1 and the feasibility of bioaugmentation for treating phenol-contaminated drinking water sources.

## Materials and Methods

### Morphology of *Acinetobacter* sp. DW-1

To examine the morphological features and characteristics of strain DW-1, scanning electron microscopy (SEM) were used. Briefly, some of the culture fluid was inoculated into fresh R2A fluid nutrient medium within several cell climbing slices (Wohong, Shanghai, China) and statically cultured at 37°C. Subsequently, cell climbing slices were removed from the R2A fluid nutrient medium at 12 h and 24 h. Then, cells grown on the cell climbing slices were fixed with 3% (v/v) glutaraldehyde at 4°C for 5 h, dehydrated in a graded ethanol series, and tert-butyl alcohol was added. The cells were dried, coated with gold, and observed using an S-3000N SEM (Hitachi, Tokyo, Japan).

### *Acinetobacter* sp. DW-1 Immobilization

Polyhedral hollow polypropylene spheres are usually applied to immobilize microorganisms in biological pre-treatment processes in drinking water treatment plants (DWTPs). Sterilized PHPSs (Jingying, Yixing, China) were added to 200 mL of mineral salt medium (MSM), and 4 mM of phenol was added as the sole carbon and energy source. The composition of the MSM was described in our previous study (Gu et al., 2018). Strain DW-1 was incubated in the MSM at 30°C without any motion for 72 h as described previously (Gu et al., 2017).

### Nanopore Sequencing, *de novo* Assembly, and Annotation

The genome of *Acinetobacter* sp. DW-1 was sequenced using the nanopore sequencing technology platform of the Biomarker Technologies Corporation (Beijing, China). After filtering, the data were assembled using the Canu v1.5 software (Koren et al., 2017). Subsequently, the genome was predicted. The Prodigal software (version 2.50) was used to predict the coding regions (Hyatt et al., 2010). Meanwhile, the tRNA scan-SE (version 1.3.1) (Lowe and Eddy, 1997), Infernal (version 1.1) (Nawrocki and Eddy, 2013), and Rfam (version 12.0) (Nawrocki et al., 2015) software packages were used to predict the non-coding RNA. In addition, the RepeatMasker software (version 4.0.5) (Tarailo-Graovac and Chen, 2009) was used to predict repeat sequences. The predicted genes were compared against the COG, GO, KEGG, Pfam, SwissProt, TrEMBL, and Nr databases using BLAST (version 4.0.5) (Ma W. et al., 2020). Raw data have been submitted to the NCBI Sequence Read Archive (SRA) under project PRJNA731647 with accession numbers SAMN19292286.

### PHPS Filter Construction

To evaluate the stability of the phenol-degrading bacterium *Acinetobacter* sp. DW-1, a BEPHPS filter was constructed, in which two organic glass columns (inner diameter = 20 cm) were filled with PHPS. One column was filled with sterilized PHPS and defined as the control check (CK) group, while the other column contained immobilized PHPS and was defined as the treatment (T) group. Raw water in batches was collected from a DWTP (surface water as source water) along the Pearl River in Guangzhou, and an additional 500 μg/L of phenol was pumped into the glass columns using peristaltic pumps at a flow rate of 4 mL/min, with an empty bed contact time (EBCT) of 20 min. The PHPS filters were operated continuously for 56 days at room temperature. The PHPSs were collected, respectively, from the two organic glass columns at the sampling ports T (top of the PHPS filters), M (middle of the PHPS filters), and B (bottom of the PHPS filters) every 7 days for biofilm observation, adenosine triphosphate (ATP) examination, phenol hydroxylase gene quantification, and microbial community analysis. The sampling ports are shown in Figure 1. Meanwhile, influent and effluent water samples from the experimental apparatus were also collected for water quality determination. For PHPS samples, only one sample was taken from each sampling port. Samples from the sampling ports T, M, and B, respectively, represent three replicate samples. While for water samples, three replicate samples were also collected.

### Biofilm Visualization and ATP Examination

The PHPSs obtained from the PHPS filter were examined using an S-3000N SEM (Hitachi, Tokyo, Japan). Before SEM observations, the immobilized PHPS was pre-treated as described in section “Morphology of *Acinetobacter* sp. DW-1.” The bioactivity of the biofilm on the PHPS was examined using a LIVE/DEAD^®^ BacLight^TM^ Bacterial Viability and Counting Kit (Invitrogen, Carlsbad, CA, United States). Dead cells were stained with PI, while living cells were dyed with SYTO9. The dyed PHPSs were observed using confocal laser scanning microscopy (CLSM) (Zeiss, Berlin, Germany). The ATP levels were examined as described in a previous study (Zhang et al., 2011).

### Water Quality Analyses

The water samples collected from the BEPHPS filters were analyzed for ammonia-nitrogen (NH_3_-N), total organic carbon (TOC), and phenol. For NH_3_-N and TOC, detection was conducted according to the national drinking water standard (GB/T 5750-2006; China). Phenol was analyzed as described previously (Gu et al., 2016).

### Denaturing Gradient Gel Electrophoresis and 16s rDNA Amplicon Sequencing

To investigate the microbial community structure on PHPSs, PCR-denaturing gradient gel electrophoresis (DGGE) analyses were conducted. Total DNA was extracted from the immobilized PHPS according to the instructions of the FastDNA SPIN Kit for Soil (MP Biomedicals, CA, United States). The 16S rRNA gene was amplified for the DGGE experiment using the primers 341f, 341f-GC, and 518r, which target the V3 region (Wang C. C. et al., 2019). PCR amplification and DGGE were performed as described previously (Gu et al., 2016). The 16S rRNA gene was amplified using the universal primers 341F and 806R, which target the V3 + V4 region with different barcodes for each sample (Wu et al., 2015). PCR amplification was conducted as described previously (Walters et al., 2016). The PCR amplification protocol was as follows: 95°C for 3 min; 27 cycles of 95°C for 30 s, 55°C for 30 s, and 72°C for 45 s; and 72°C for 10 min. Subsequently, the PCR products were sequenced using an Illumina HiSeq 2500 platform (Illumina, San Diego, CA, United States). The reads from the original DNA fragments were merged using FLASH^1^ (Magoc and Salzberg, 2011), all sequences were clustered into operational taxonomic units (OTUs) at 97% sequence similarity using Mothur version 1.34.0^2^ (Hou et al., 2018b). A representative sequence for each OTU was selected, and a Ribosomal Database Project (RDP) classifier was used to assign taxonomic identity to each representative sequence (Deakin et al., 2018). The species annotation of representative sequences was conducted using VSEARCH based on the SILVA 132 database. QIIME and R package were employed to analyze the sequencing data as described previously (Wu et al., 2020). In addition, FAPROTAX was used to predict the biochemical cycle of environmental samples (Li C. et al., 2020).

### Quantification of Phenol Hydroxylase Gene

Quantitative real-time PCR was used to analyze the abundance of phenol hydroxylase (PH) gene. Plasmids pUC57 carrying PH gene (Supplementary Table 3) of *Acinetobacter* sp. DW-1 were used for standard analysis. PH gene was amplified using the primers sets: Lphf (5′-CGCCAGAACATTTATCGATC-3′), Lphr (5′-AGGCATCAAGATCACCGACTG-3′) (Xu et al., 2001). PCR amplification conditions were performed as previously described (Gu et al., 2017).

## Results and Discussion

### Morphological Observations of Strain DW-1

**FIGURE 2:**
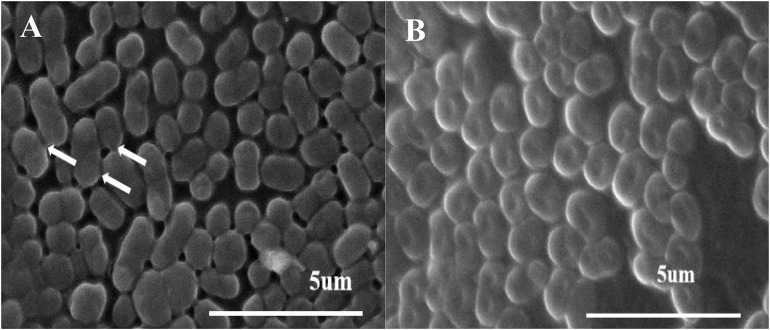
SEM micrographs of strain DW-1 after incubation for 12 h **(A)** and 24 h **(B)**.

Scanning electron microscopy observations showed that, after incubating for 12 h, the cells grew densely on the cell climbing slices. Anchor-like appendages were clearly visible between the cells, which might make the connections closer, this characteristic was observed when strain DW-1 cultured in fluid nutrient medium in our previous study (Gu et al., 2017). Moreover, this characteristic may be related to the adhesion ability of strain DW-1 (Figure 2A). In addition, after incubation for 24 h, strain DW-1 grew well on solid surfaces, with cells arranged side by side (Figure 2B). This also indicates the excellent biofilm-forming ability of strain DW-1. Therefore, strain DW-1 is a good biological material for cell immobilization.

### Genome Analysis

**FIGURE 3:**
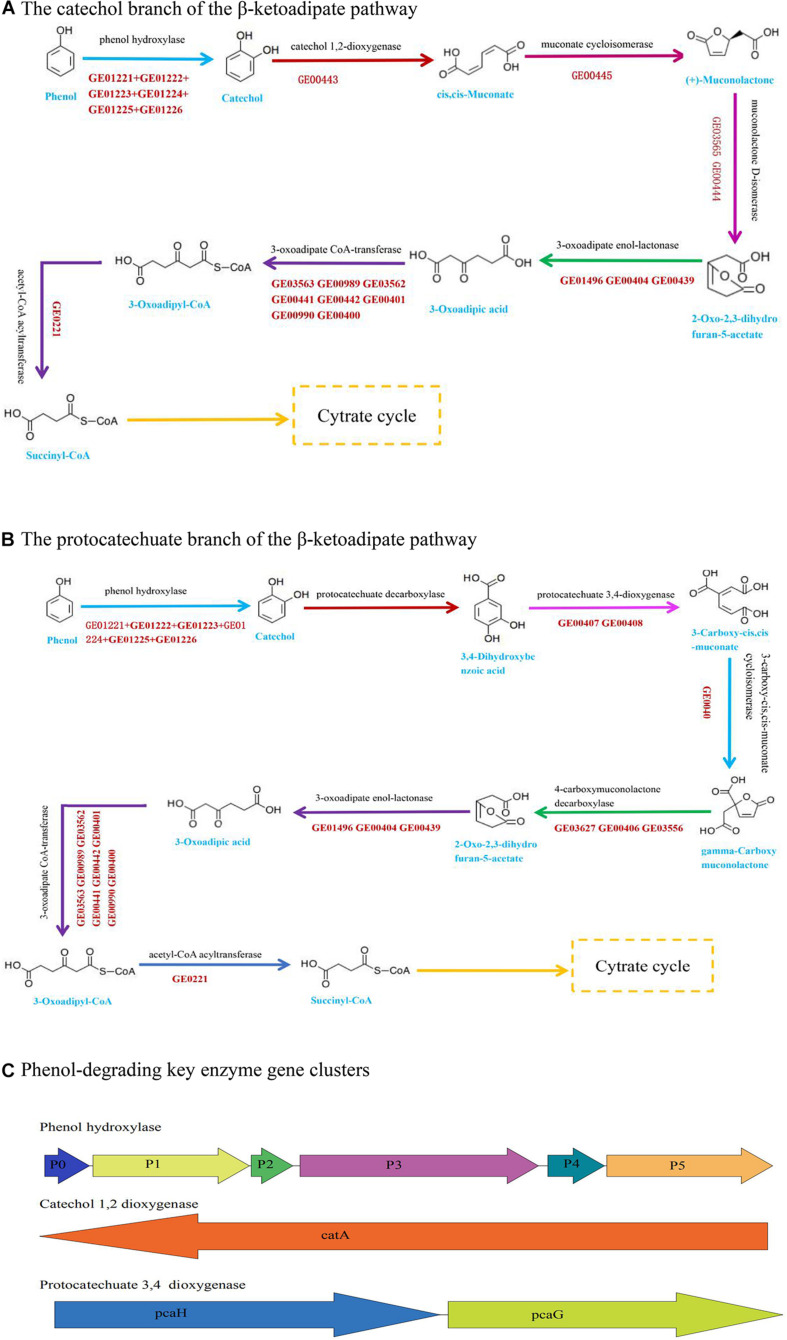
Pathway and key enzyme gene clusters involved in phenol degradation based on genome data. **(A)** The catechol branch of the β-ketoadipate pathway in Acinetobacter sp. DW-1. **(B)** The protocatechuate branch of the β-ketoadipate pathway in *Acinetobacter* sp. DW-1. **(C)** Genomic organization of the key enzyme gene clusters in phenol degradation.

The nanopore sequencing data showed that the total genome size of *Acinetobacter* DW-1 was 4.13 Mb. Contigs were then assembled into two scaffolds with an average length of 4,134,434 bp. The G + C content of the genome was 38.78%. A total of 3,859 unigenes were obtained, with an average length of 933 bp. The information of the annotated genes in functional databases were shown in Supplementary Table 1 and Supplementary Figure 2. Previous studies have shown that *Acinetobacter* sp. are able to degrade large amounts of organic matter, including phenol (Wang et al., 2007), malathion (Xie et al., 2009), methyl parathion (Liu et al., 2007), chloroanilines (Hongsawat and Vangnai, 2011), and aniline (Hou et al., 2018a). The KEGG_annotation also indicated that *Acinetobacter* DW-1 is able to metabolize benzoate and fluorobenzoate. Furthermore, in the benzoate pathway, as can be seen from Figure 3A, we found that the most important phenol catabolic enzymes in the catechol branch of the ortho-cleavage pathway include phenol hydroxylase (EC:1.14.13), catechol 1, 2-dioxygenase (EC:1.13.11.1), muconate cycloisomerases (EC:5.5.1.1), muconolactone D-isomerase (EC:5.3.3.4), 3-oxoadipate enol-lactonase (EC:3.1.1.24), 3-oxoadipate CoA-transferase (EC:2.8.3.6), acetyl-CoA acyltransferase (EC:2.3.1.16), and 3-oxoadipyl-CoA thiolase (EC:2.3.1.174). Meanwhile, the key enzyme gene clusters of phenol hydroxylase and catechol 1, 2-dioxygenase was also annotated (Figure 3C). In addition, *Acinetobacter* DW-1 was found to metabolize phenol by the protocatechuate branch of the β-ketoadipate pathway (Figure 3B), in which key metabolic enzymes were also observed, including protocatechuate 3, 4-dioxygenase (EC:1.13.11.3), 3-carboxy-cis, cis-muconate cycloisomerase (EC:5.5.1.2), and 4-carboxymuconolactone decarboxylase (EC:4.1.1.44). Similarly, the key enzyme gene clusters of protocatechuate 3, 4-dioxygenase were also found, as shown in Figure 3C. Therefore, we assumed that *Acinetobacter* DW-1 has the ability to utilize phenol by both the catechol and protocatechuate branches of the β-ketoadipate pathway, which is consistent with the transcriptome data analysis in our previous study (Gu et al., 2017). However, some proteins involved in phenol degradation through the meta-pathway were upregulated, but the key enzyme (catechol 2, 3-dioxygenase) involved in the meta-cleavage pathway was not observed. In addition, the genes of catechol 2, 3-dioxygenase were not annotated (Gu et al., 2017). Thus, the phenol metabolic pathway in *Acinetobacter* DW-1 requires further confirmation.

### Biofilm Observation

**FIGURE 4:**
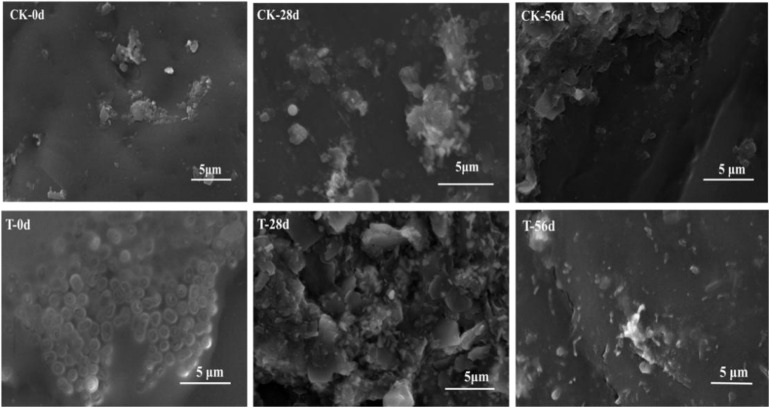
Biofilms on the PHPS observed under SEM (CK, control check; T, treatment).

**FIGURE 5:**
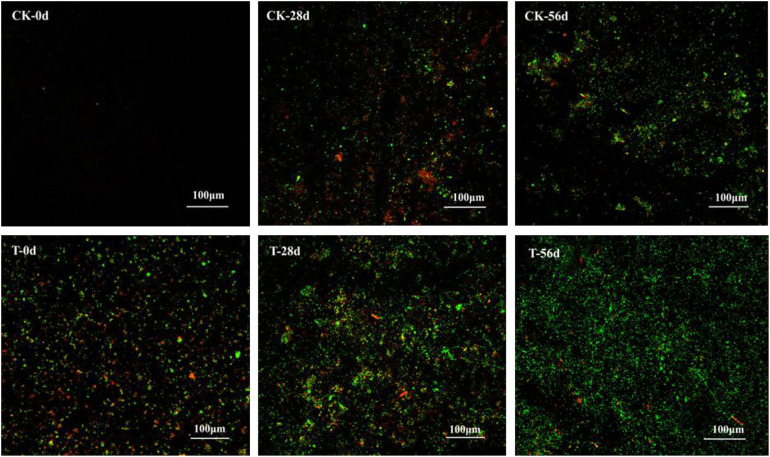
Biofilm on the PHPS observed under CLSM. The fluorescence of which includes PI (red) for dead cells, and SYTO9 (green) for living cells. The bars represent 100 μm.

**FIGURE 6:**
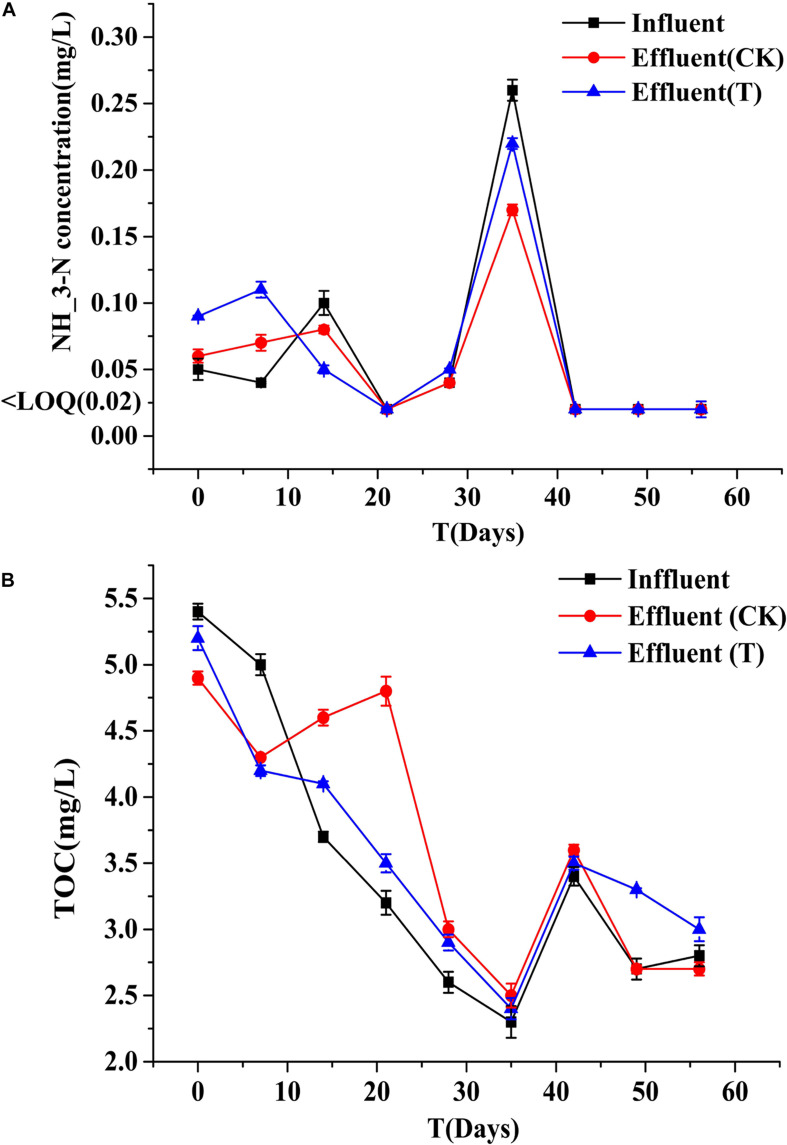
NH_3_-N **(A)** and TOC **(B)** concentrations of the influent and effluents. Error bars indicate standard deviation (*n* = 3).

Biofilms developed on the PHPS that were retrieved from the CK and T groups of the PHPS filters were observed under SEM, as shown in Figure 4. On day 0, for the T group, the *Acinetobacter* sp. DW-1 cells were side by side on the PHPS surfaces. When the BEPHPS filter was run for 28 days, the microbes and their extracellular secretions accumulated on the PHPS surfaces in the T group. A few bacteria adhered to the PHPS surfaces in the CK group. In addition, algae appeared on the PHPS surfaces at day 42 in both the CK and T groups (Supplementary Figure 3). After the PHPS filters was run for 56 days, the biofilm became thicker and denser, and fully covered the PHPS surfaces in both the CK and T groups. The growth of algae requires large amounts of nitrogen and phosphorus; therefore, the surfaces of the PHPS might have enriched a variety of nutrients. The biofilm on the PHPS surfaces grew into agglomerates and flocculates, which formed a thicker bio-colloid. In addition, Figure 5 shows that the bioactivity of the biofilm on the PHPS gradually increased in both the CK and T groups, and the bioactivity peaked on day 56, which also occurred in the biofilm observed by SEM. ATP determination is an effective method that reflects the bioactivity of bacteria (Zhang et al., 2013). At the initial stage (0 day), bioactivity in the CK group was lowest, whereas it was relatively high in the T group. This results were consistent with observation results by SEM and CLSM. From day 0 to day 42 of the operation period, the bioactivity increased gradually in the effluent water from both the CK and T groups (Supplementary Figure 4). After day 42, the bioactivity did not increase, and even decreased in the CK group. During the entire 56 days operational period, the bioactivity of the CK group increased from 4.68626E-9 to 2.00743E-7 mol/g. The bioactivity of the T group increased from 2.8122E-7 to 6.64253E-7 mol/g. On day 56 of the PHPS filter operation, the bioactivity of the CK group and T group reached a peak, the results were consistent with the CLSM observations. As shown in Figure 6A, the NH_3_-N concentration was a function of time when the PHPS filter was applied. During the initial stage, the NH_3_-N concentration of the T group’s effluent water was higher than that of the CK group. After 20 days of continuous operation, the NH_3_-N concentration of the influent water was higher than those of the CK and T effluents, which indicates that the PHPS filter effectively removed NH_3_-N. This might be attributed to increased bioactivity. In addition, Figure 6B shows that the TOC concentrations of the influent water were higher for the first 10 days than those of the CK and T effluents, indicating that the PHPS filter effectively removed organic matter during the initial stage. However, from day 10 to day 28 the removal efficiency was not good, which might have been due to the separation of the unstable biofilm growth on the PHPS. Furthermore, phenol was not detected in T effluents throughout the water treatment process, while after 35 days, phenol could be completely removed in the CK group (Supplementary Figure 5). Thus, the PHPS filters has the potential to remove NH_3_-N and TOC, particularly phenol.

### Stability Analysis of Phenol-Degrading Bacteria

**FIGURE 7:**
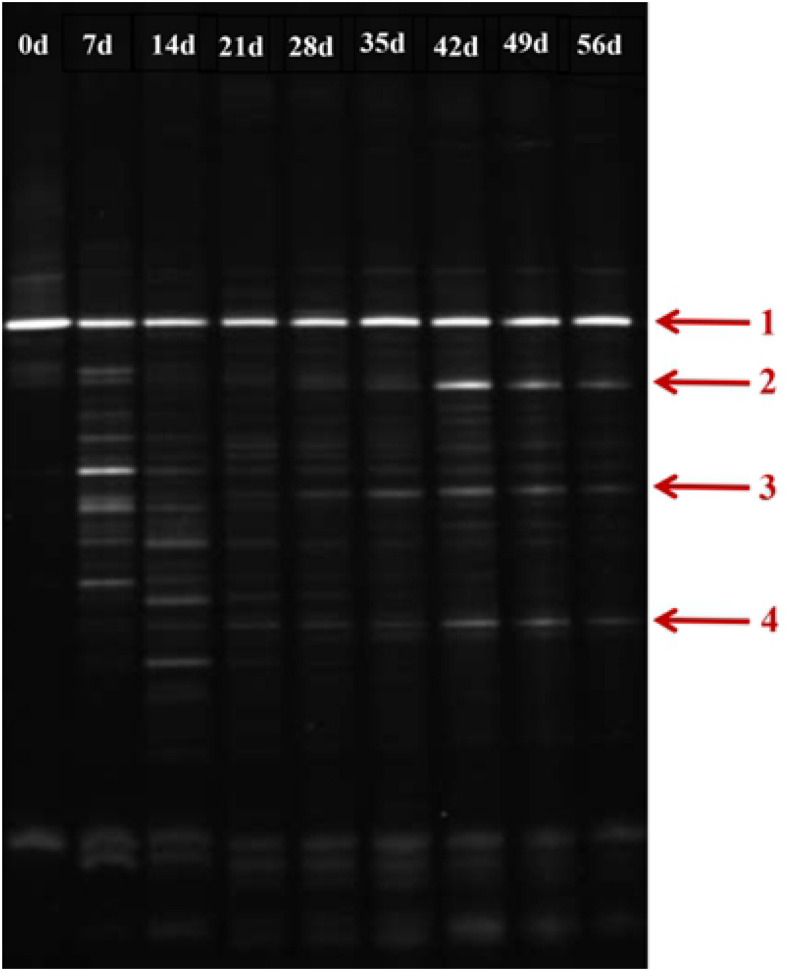
DGGE profile of the biofilm on the PHPS during the 56 days water treatment process (Arrow 1 represents *Acinetobacter* sp. DW-1).

**FIGURE 8:**
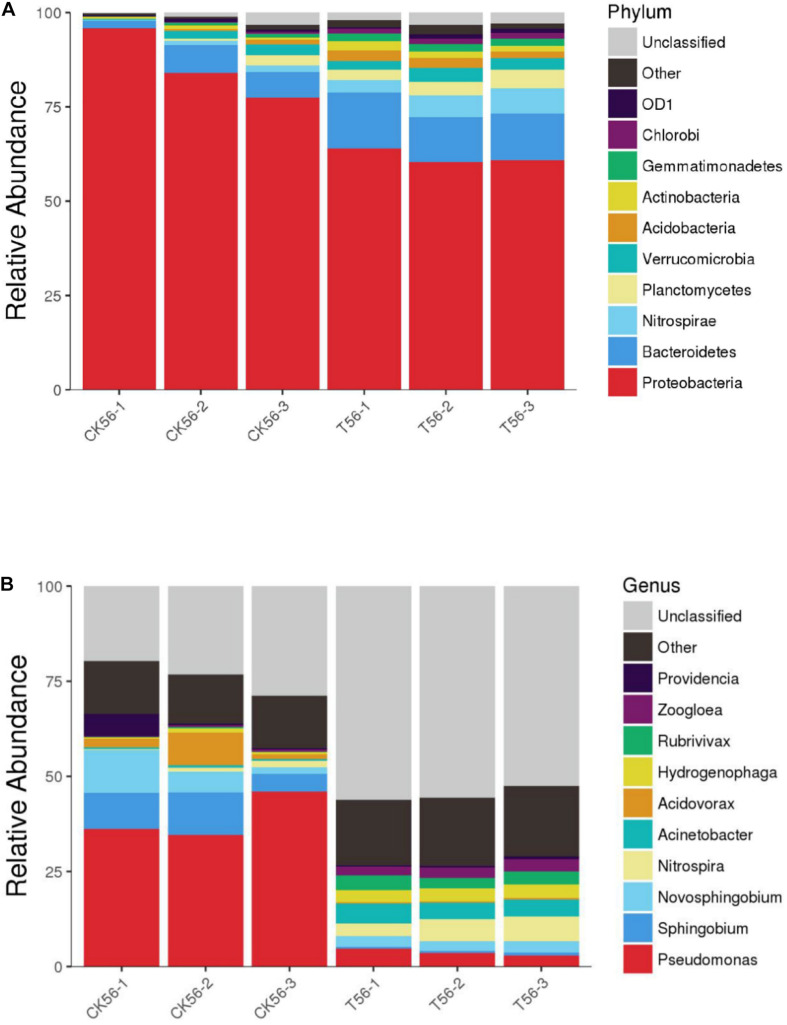
Diversity and composition of the bacterial community in the PHPS filter at the phylum **(A)** and genus **(B)** level on day 56. Phyla or genera with relative abundances of less than 0.1% were classified as “other.”

As shown in Figure 7, during the initial stage of the BEPHPS filter operation (day 0), only band 1 was present, namely *Acinetobacter* sp. DW-1. However, by day 42, three indigenous bacteria (bands 2, 3, and 4) had invaded the bacterial community on the PHPS and persisted in the BEPHPS filter. The three invasive bacteria, along with band 1, became the dominant bacteria during the final stage of the BEPHPS filter operation. Band 1 was always the predominantly influential bacteria throughout the entire BEPHPS filter operation. These results indicated that *Acinetobacter* sp. DW-1 was highly stable in a complex water environment. We also used 16S rRNA gene amplicon sequencing to investigate the stability of the seed bacteria and the bacterial community diversity. The results also indicated that *Acinetobacter* sp. was highly abundant in the T group on day 21 (Supplementary Figure 7), even on day 56 (Figures 8A,B). Especially, on day 21, the relative abundance of *Acinetobacter* sp. could still account for more than 98% in the T group. However, on day 56, the relative abundance of *Acinetobacter* sp. was reduced, but it still maintained a certain high ratio, along with *Pseudomonas* sp., *Nitrospira* sp., *Rubrivivax* sp., and *Hydrogenophaga* sp. in the T group. In addition, the quantification of PH gene results (Supplementary Figure 8) showed that *Acinetobacter* sp. DW-1 also kept a high amount in the T group, although the absolute abundance of PH gene gradually decreased during water treatment process. However, it was still higher than that in the CK group. Thus we can see that results of PGGE, 16S rRNA gene amplicon sequencing and qPCR of PH gene corroborated each other. Previous studies have shown that *Pseudomonas* sp. can degrade various organic substances, including butachlor (Duc et al., 2020), pyrene (Li J. et al., 2020), para-xylene (Li H. et al., 2020) and di-n-butyl phthalate (Osorio et al., 2020), *Nitrospira* sp. can effectively remove NH_3_-N (Gorre and Himabindu, 2014; Liu et al., 2017), which was related to high NH_3_-N removal efficiency after 35 days of continuous operation. *Rubrivivax* sp. can tolerate high concentrations of aniline (Mujahid et al., 2015), and *Hydrogenophaga* sp. can degrade 2-butoxyethanol (Woiski et al., 2020) and might be responsible for microcystin degradation (Ding et al., 2020). In contrast, in the CK group both on day 21 and 56, the predominant bacteria were *Pseudomonas* sp., *Sphingobium* sp., *Novosphingobium* sp., and *Acidovorax* sp. *Sphingobium* sp. were reported to degrade Bisphenol A (Jia et al., 2020), carbofuran (Jiang et al., 2020) and 17 Archetypal Organophosphate (Azam et al., 2019). *Novosphingobium sp.* is involved in the degradation of microcystin (Wang et al., 2020), antibiotics (Zeng et al., 2020), and beta-estradiol (Li S. et al., 2020), *Acidovorax* sp. degrades BTEX (Benedek et al., 2018), phenanthrene (Singleton et al., 2018), and 1, 2-dichlorobenzene (Cui et al., 2017). At the phylum level, we found that the dominant bacteria in the CK group were *Proteobacteria*, followed by *Bacteroidetes*. Previous studies have shown that *Proteobacteria* are generally dominant in drinking water sources (Hou et al., 2018b; Han et al., 2020), and some members of *Proteobacteria* are competitive at low-nutrient concentrations (Liao et al., 2013; Lautenschlager et al., 2014). This phylum has been regarded as highly stable and is not affected by water treatment processes or seasonal changes; thus, they can exist in drinking water biofilters for an extended period and are abundant (de Vera et al., 2018). This might indicate why *Proteobacteria* were dominant in both the CK and T groups. However, we found that the overall microbial community structure of the CK group was different from that of the T group. Microbial community structures in both natural and man-made ecosystems are particularly vulnerable. Initially, the composition of the “seed” microbe and the unique lifestyles of microorganisms are important for determining the bacterial community composition, especially the complex ecosystem involved in multiple environmental niches (Ma B. et al., 2020). Thus, the “seed” *Acinetobacter* sp. DW-1 bacteria might affect the microbial community composition of the PHPS filter, which could lead to a different biofilm-forming assembly between natural formation and manual intervention. As the abundance of ecological functions in microorganisms determines their pollutant removal performance, Functional Annotation of Prokaryotic Taxa (FAPROTAX) ecological function can provide further insight into the pollutant biodegradation process. As seen from Supplementary Figure 6, the most abundant functional group in the CK group was chemoheterotrophs (38.3%), followed by aerobic chemoheterotrophs (35.4%), fermenters (2.0%), and methylotrophs (1.3%). The most abundant functional group in the T group was chemoheterotrophs (15.9%), followed by aerobic chemoheterotrophs (14.3%), aromatic compound degraders (3.0%), aerobic nitrite oxidizers (2.6%), and nitrifiers (2.7%). Detailed information on the functional groups is provided in Supplementary Table 2. The results indicated that the CK and T groups were effective for pollutant removal, and the T group was suitable for the degradation of a variety of pollutants. Therefore, the microbial community structure could affect the ecological function.

## Conclusion

In this study, the whole genome of *Acinetobacter* sp. DW-1 was sequenced using third generation sequencing technology. The genomic information suggests that *Acinetobacter* sp. DW-1 degrade phenol by the catechol and protocatechuate branches of the β-ketoadipate pathway. PHPS filters operated for 56 days indicated that the bioactivity of the biofilm on the PHPS gradually increased in both the CK and T groups, and peaked on day 56, which resulted in a good NH_3_-N removal efficiency, and they have the potential to remove TOC, particularly phenol. In addition, except for band 1 (namely *Acinetobacter* sp. DW-1), three other indigenous bacteria (bands 2, 3, and 4) dominated in the BEPHPS filter on day 56. 16S rRNA gene amplicon sequencing results indicated that *Acinetobacter* sp., along with *Pseudomonas* sp., *Nitrospira* sp., *Rubrivivax* sp. were the predominant bacteria in the BEPHPS filter. The quantification of phenol hydroxylase gene results showed that *Acinetobacter* sp. also kept a high amount in the T group. The above results suggest that *Acinetobacter* sp. DW-1 could be competitive and maintain stability during actual water treatment. In addition, the seeded bacteria could also affect the microbial community structure on immobilized materials. This study advances the current understanding of the phenol degradation mechanism of *Acinetobacter* sp. DW-1 isolated from oligotrophic environment, as well as provides new insights into the stability of seeded bacteria as well as microbial interaction during drinking water treatment. These findings provide theoretical support for the practical application of functional microbe bioaugmentation in the pretreatment of micro-polluted drinking water source, and genetic information for modification of phenol efficient engineering strain in the future work.

## Data Availability Statement

The data presented in the study are deposited in the NCBI Sequence Read Archive repository, accession number SRR14689246.

## Author Contributions

QW and QG conceived and designed the experiments. QG, LW, and MS performed the experiments. QG, HW, YZ, and XW analyzed the data. QW, YD, JW, WG, and JZ contributed to reagents, materials, and analysis tools. QG wrote the manuscript. MC, LX, and QG reviewed the manuscript. All authors contributed to the article and approved the submitted version.

## Conflict of Interest

The authors declare that the research was conducted in the absence of any commercial or financial relationships that could be construed as a potential conflict of interest.

## Footnotes

1 http://ccb.jhu.edu/software/FLASH/

2 https://mothur.org
