# Supplementary material for: Genomic Analysis and Stability Evaluation of the Phenol-Degrading Bacterium Acinetobacter sp. DW-1 During Water Treatment
Source: Front Microbiol. 2021 Jul 13;12:687511. doi: 10.3389/fmicb.2021.687511 (PMC8313972; doi:10.3389/fmicb.2021.687511)
Supplement: Supplementary file 1 [file Data_Sheet_1.docx]

***Supplementary Material***

**Genome analysis and stability evaluation of the phenol-degrading bacterium-*Acinetobacter* sp. DW-1 during water treatment process**

Qihui Gu^1,#^, Moutong Chen^1,#^, Jumei Zhang^1^, Weipeng Guo^1^, Huiqing Wu^1^, Ming Sun^1^, Lei Wei ^1^, Juan Wang^2^, Xianhu Wei^1^, Youxiong Zhang^1^, Qinghua Ye^1^, Liang Xue^1^, Rui Pang^1^, Yu Ding^3,^*, Qingping Wu^1,^*

*** Correspondence:** Qingping Wu: E-mail: [wuqp203@163.com](mailto:wuqp203@163.com)

**Supplementary Figures**


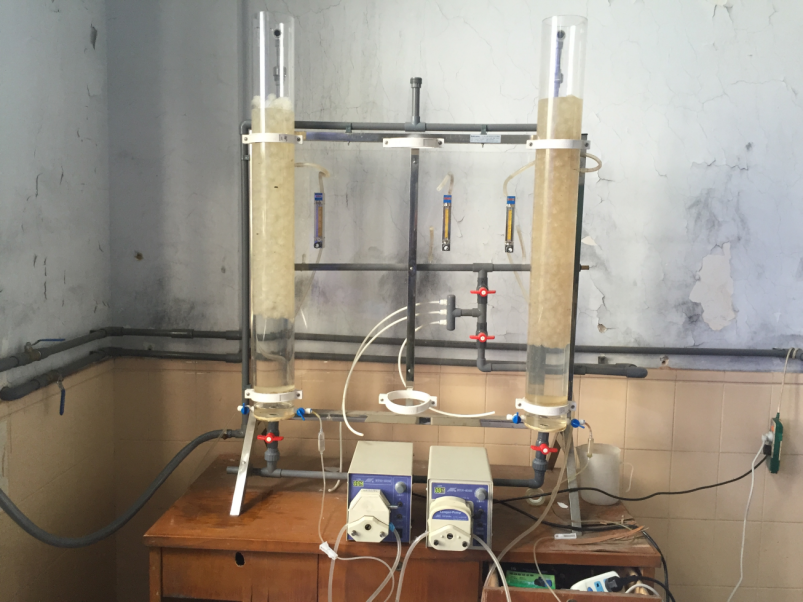


Fig.S1 Physical diagram of BEPHPS filter used for continuous experiments.


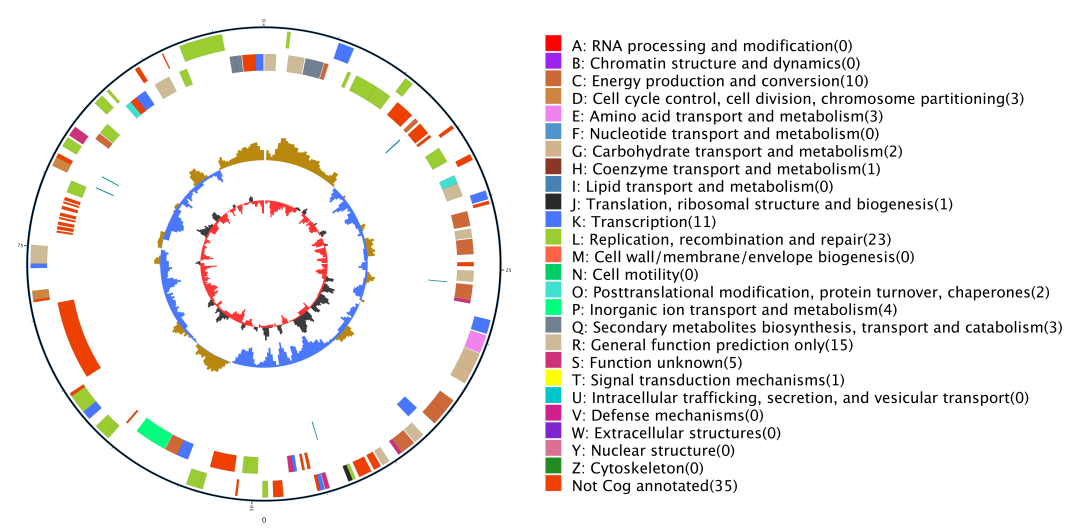


Fig. S2 Genomic circle map of *Acinetobacter* sp. DW-1


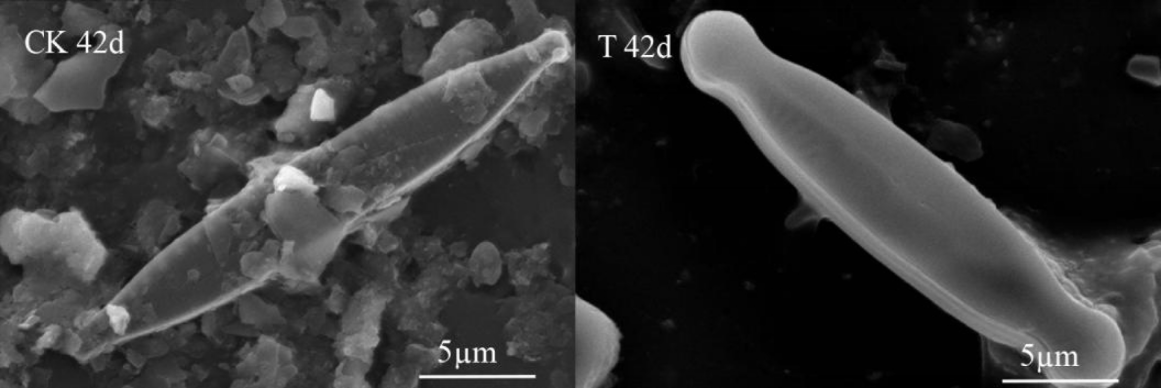


Fig.S3 Biofilm on PHPS visualized by scanning electron microscopy. PHPS samples were taken on the 42th day of PHPS filter operation. Note:CK,control check; T,treatment.


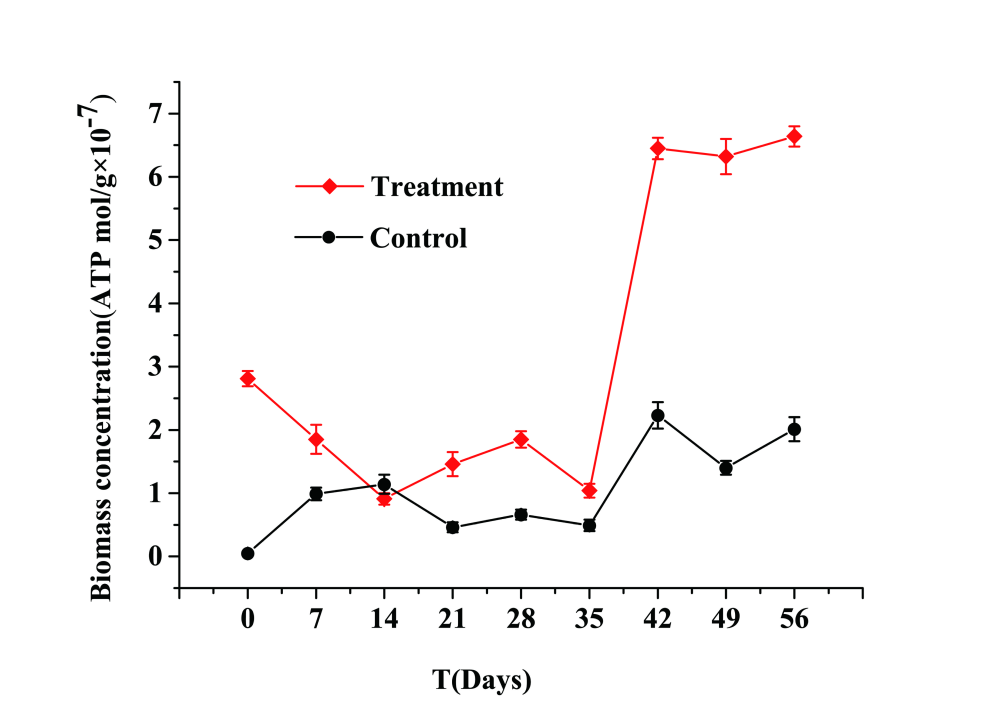


Fig.S4 ATP determination on the PHPS. Error bars indicate standard deviation (n = 3).


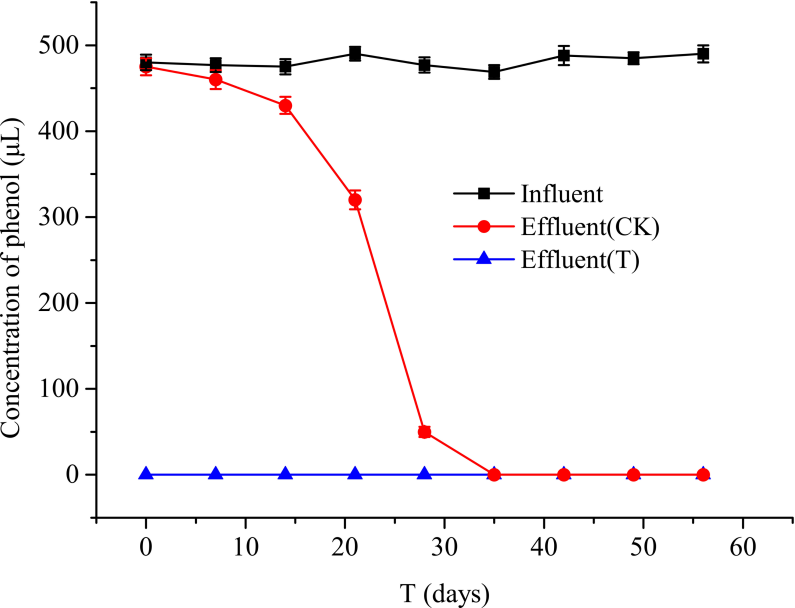


Fig.S5 Phenol concentrations of the influent and effluents. Error bars indicate standard deviation(n=3).


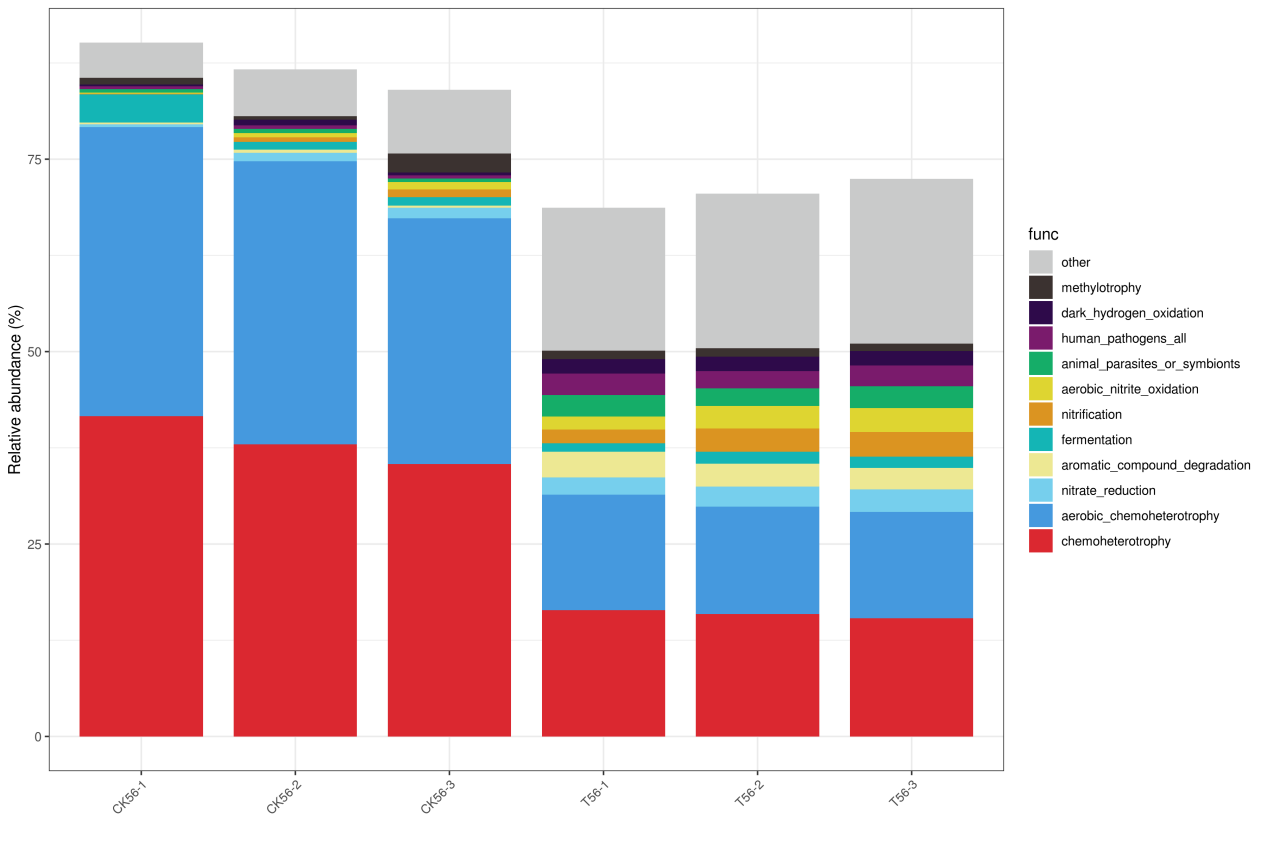


Fig. S6 FAPROTAX ecological function predictions of the CK and T group samples.





Fig.S7 Diversity and composition of the bacterial community in the PHPS filter at the phylum (A) and genus (B) level on day 21. Phyla or genera with relative abundances of less than 0.1% were classified as “other”.


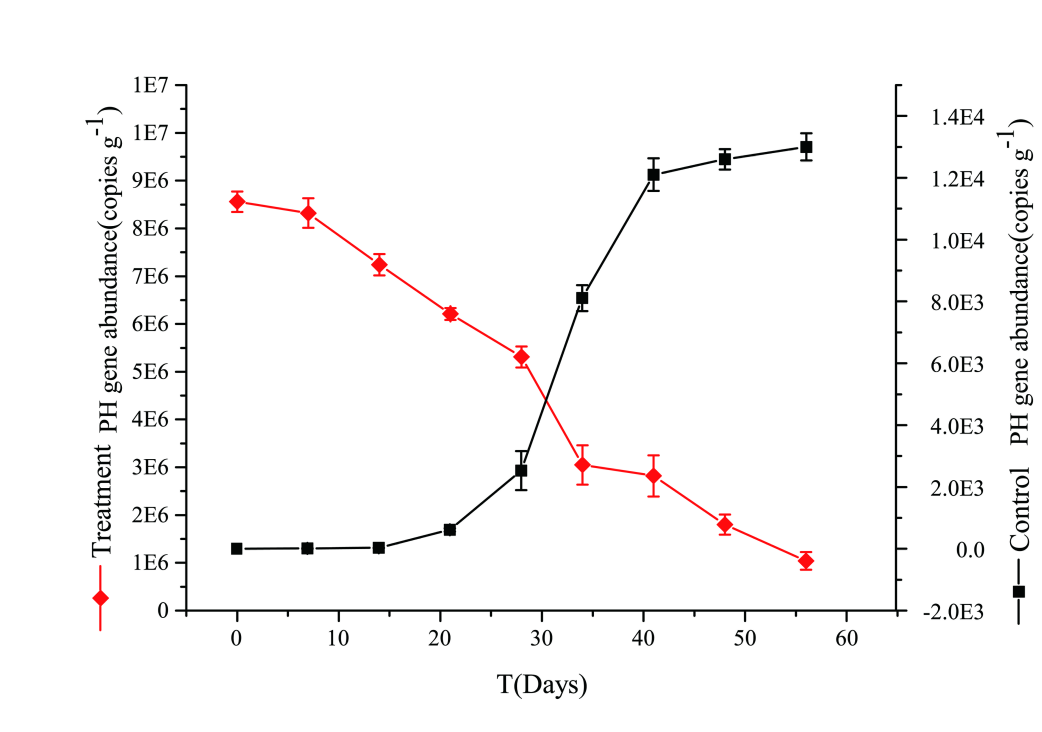


Fig.S8 Absolute abundance of phenol hydroxylase gene on the PHPS. Error bars indicate standard deviation (n = 3)

**Supplementary Tables**

Table S1 Gene function annotation of *Acinetobacter* sp. DW-1

| **Database** | **Number** | **100≤length<300** | **length≥300** |
| --- | --- | --- | --- |
| COG_Annotation | 2,854 | 1,357 | 1,401 |
| GO_Annotation | 2,531 | 1,127 | 1,321 |
| KEGG_Annotation | 1,925 | 845 | 1,014 |
| Pfam_Annotation | 3,161 | 1,516 | 1,527 |
| Swissprot_Annotation | 2,660 | 1,199 | 1,380 |
| TrEMBL_Annotation | 3,858 | 1,886 | 1,644 |

Table S2 Display of functional prediction results by Functional Annotation of Prokaryotic Taxa (FAPROTAX)

| Function | CK56-1 | CK56-2 | CK56-3 | T56-1 | T56-2 | T56-3 |
| --- | --- | --- | --- | --- | --- | --- |
| methanotrophy | 0 | 0.00289821469974496 | 0.00722491149483419 | 0.032351553548561 | 0.0196113039556 | 0.0270336980923612 |
| acetoclastic_methanogenesis | 0 | 0 | 0 | 0 | 0 | 0.00587689088964374 |
| methanogenesis_by_disproportionation_of_methyl_groups | 0 | 0 | 0 | 0 | 0 | 0 |
| methanogenesis_using_formate | 0 | 0 | 0 | 0 | 0 | 0 |
| methanogenesis_by_CO2_reduction_with_H2 | 0 | 0 | 0 | 0 | 0 | 0 |
| methanogenesis_by_reduction_of_methyl_compounds_with_H2 | 0 | 0 | 0 | 0 | 0 | 0 |
| hydrogenotrophic_methanogenesis | 0 | 0 | 0 | 0 | 0 | 0 |
| methanogenesis | 0 | 0 | 0 | 0 | 0 | 0.00587689088964374 |
| methanol_oxidation | 0.858724843340738 | 0.450672385810341 | 2.45935987284156 | 1.04872952753252 | 1.0884273695358 | 0.92032111331821 |
| methylotrophy | 0.858724843340738 | 0.453570600510086 | 2.46658478433639 | 1.08108108108108 | 1.1080386734914 | 0.947354811410571 |
| aerobic_ammonia_oxidation | 0.00663107987135705 | 0.0217366102480872 | 0.0419044866700383 | 0.0727909954842623 | 0.10982330215136 | 0.0916794978784424 |
| aerobic_nitrite_oxidation | 0.106097277941713 | 0.560804544400649 | 0.962358211111914 | 1.6876727101166 | 2.91816202859328 | 3.1194536842229 |
| nitrification | 0.11272835781307 | 0.582541154648736 | 1.00426269778195 | 1.76046370560086 | 3.02798533074464 | 3.21113318210134 |
| sulfate_respiration | 0 | 0 | 0.00433494689690051 | 0.0121318325807104 | 0 | 0.00940302542342999 |
| sulfur_respiration | 0 | 0 | 0 | 0 | 0 | 0 |
| dark_sulfite_oxidation | 0 | 0 | 0 | 0 | 0 | 0 |
| sulfite_respiration | 0.0265243194854282 | 0.00579642939948991 | 0.00722491149483419 | 0.00808788838714026 | 0.00588339118668 | 0.0305598326261475 |
| thiosulfate_respiration | 0.0265243194854282 | 0.00579642939948991 | 0.00722491149483419 | 0.00808788838714026 | 0.00588339118668 | 0.0305598326261475 |
| respiration_of_sulfur_compounds | 0.0265243194854282 | 0.00579642939948991 | 0.0115598583917347 | 0.0202197209678506 | 0.00588339118668 | 0.0399628580495775 |
| arsenate_detoxification | 0.0232087795497497 | 0.0144910734987248 | 0 | 0.00404394419357013 | 0.00392226079112 | 0.0023507563558575 |
| arsenate_respiration | 0 | 0 | 0 | 0 | 0 | 0 |
| dissimilatory_arsenate_reduction | 0.0232087795497497 | 0.0144910734987248 | 0 | 0.00404394419357013 | 0.00392226079112 | 0.0023507563558575 |
| arsenite_oxidation_detoxification | 0 | 0 | 0 | 0 | 0 | 0 |
| arsenite_oxidation_energy_yielding | 0 | 0 | 0 | 0 | 0 | 0 |
| dissimilatory_arsenite_oxidation | 0 | 0 | 0 | 0 | 0 | 0 |
| anammox | 0 | 0 | 0 | 0 | 0 | 0 |
| nitrate_denitrification | 0.109412817877391 | 0.375318803616972 | 0.378585362329311 | 1.4261643189324 | 1.57478770763468 | 1.7172275179539 |
| nitrite_denitrification | 0.109412817877391 | 0.375318803616972 | 0.378585362329311 | 1.4261643189324 | 1.57478770763468 | 1.7172275179539 |
| nitrous_oxide_denitrification | 0.109412817877391 | 0.375318803616972 | 0.378585362329311 | 1.4261643189324 | 1.57478770763468 | 1.7172275179539 |
| denitrification | 0.109412817877391 | 0.375318803616972 | 0.378585362329311 | 1.4261643189324 | 1.57478770763468 | 1.7172275179539 |
| chitinolysis | 0.00663107987135705 | 0 | 0 | 0.0269596279571342 | 0.00588339118668 | 0.0270336980923612 |
| knallgas_bacteria | 0.0165776996783926 | 0.036227683746812 | 0.0202297521855357 | 0.0188717395699939 | 0.01176678237336 | 0.0105784036013587 |
| dark_hydrogen_oxidation | 0.218825635754783 | 0.768026895432414 | 0.355465645545842 | 1.88313001280582 | 1.85915161499088 | 1.86532516837292 |
| nitrogen_fixation | 0.0530486389708564 | 0.0681080454440065 | 0.0245646990824362 | 0.517624856776976 | 0.56480555392128 | 0.590039845320232 |
| nitrate_ammonification | 0.00994661980703558 | 0.0130419661488523 | 0.0231197167834694 | 0.00134798139785671 | 0.00784452158224 | 0.0246829417365037 |
| nitrite_ammonification | 0.00994661980703558 | 0.0130419661488523 | 0.0231197167834694 | 0.0229156837635641 | 0.00784452158224 | 0.0246829417365037 |
| nitrite_respiration | 0.119359437684427 | 0.388360769765824 | 0.401705079112781 | 1.45177596549168 | 1.58263222921692 | 1.74191045969041 |
| cellulolysis | 0.0132621597427141 | 0.00289821469974496 | 0.00577992919586735 | 0.0202197209678506 | 0.01372791276892 | 0.0176306726689312 |
| xylanolysis | 0.00331553993567853 | 0.00289821469974496 | 0.00144498229896684 | 0.00269596279571342 | 0.00196113039556 | 0.00352613453378625 |
| dark_sulfide_oxidation | 0 | 0 | 0 | 0.00134798139785671 | 0 | 0.0023507563558575 |
| dark_sulfur_oxidation | 0 | 0 | 0 | 0 | 0 | 0 |
| dark_thiosulfate_oxidation | 0 | 0 | 0 | 0 | 0 | 0 |
| dark_oxidation_of_sulfur_compounds | 0.00331553993567853 | 0.00579642939948991 | 0.00144498229896684 | 0.0161757767742805 | 0.00784452158224 | 0.014104538135145 |
| manganese_oxidation | 0.0431020191638208 | 0.0579642939948991 | 0.0202297521855357 | 0.276336186560625 | 1.0982330215136 | 0.640581106971168 |
| manganese_respiration | 0 | 0 | 0 | 0 | 0 | 0 |
| ligninolysis | 0 | 0 | 0 | 0 | 0 | 0 |
| fermentation | 3.63714730943934 | 1.03466264780895 | 1.1372010692869 | 1.09321291366179 | 1.57674883803024 | 1.4433644024965 |
| aerobic_chemoheterotrophy | 37.5849607108518 | 36.7899373985625 | 31.9687883823423 | 14.9747253487902 | 13.9828597203428 | 13.8130443470187 |
| invertebrate_parasites | 0 | 0 | 0 | 0 | 0.00392226079112 | 0 |
| human_pathogens_septicemia | 0.102781738006034 | 0.036227683746812 | 0.0375695397731378 | 0.0363954977421312 | 0.05098939028456 | 0.0893287415225849 |
| human_pathogens_pneumonia | 0 | 0.00144910734987248 | 0 | 0 | 0.00196113039556 | 0 |
| human_pathogens_nosocomia | 0 | 0 | 0 | 0 | 0 | 0 |
| human_pathogens_meningitis | 0 | 0 | 0 | 0 | 0 | 0 |
| human_pathogens_gastroenteritis | 0.0265243194854282 | 0.00579642939948991 | 0.00722491149483419 | 0.00808788838714026 | 0.00588339118668 | 0.0305598326261475 |
| human_pathogens_diarrhea | 0.0265243194854282 | 0.0289821469974496 | 0.124268477711148 | 0.032351553548561 | 0.00784452158224 | 0.398453202317846 |
| human_pathogens_all | 0.371340472795995 | 0.430384882912126 | 0.411819955205549 | 2.79436543775696 | 2.25137769410288 | 2.71747434737127 |
| fish_parasites | 0 | 0 | 0 | 0 | 0 | 0 |
| human_gut | 0.0895195782633202 | 0.0840482262926038 | 0.0158948052886352 | 0.00943586978499697 | 0.01176678237336 | 0.0869779851667274 |
| mammal_gut | 0.0895195782633202 | 0.0854973336424762 | 0.0158948052886352 | 0.00943586978499697 | 0.01176678237336 | 0.0869779851667274 |
| animal_parasites_or_symbionts | 0.474122210802029 | 0.543415256202179 | 0.430604725092118 | 2.80784525173553 | 2.27295012845404 | 2.80915384524971 |
| plant_pathogen | 0.00663107987135705 | 0.0130419661488523 | 0.00144498229896684 | 0.0781829210756892 | 0.06667843344904 | 0.0658211779640099 |
| oil_bioremediation | 0 | 0 | 0 | 0 | 0 | 0 |
| aromatic_hydrocarbon_degradation | 0 | 0.00579642939948991 | 0 | 0 | 0.00588339118668 | 0 |
| aromatic_compound_degradation | 0.228772255561818 | 0.391258984465569 | 0.241312043927462 | 3.34164588528678 | 2.95542350610892 | 2.79857544164835 |
| aliphatic_non_methane_hydrocarbon_degradation | 0 | 0.00579642939948991 | 0 | 0 | 0.00588339118668 | 0 |
| hydrocarbon_degradation | 0.0132621597427141 | 0.0115928587989798 | 0.0115598583917347 | 0.0363954977421312 | 0.03530034712008 | 0.0493658834730074 |
| dark_iron_oxidation | 0 | 0 | 0 | 0 | 0 | 0 |
| iron_respiration | 0 | 0 | 0 | 0.00808788838714026 | 0 | 0.00822764724550124 |
| nitrate_respiration | 0.175723616590962 | 0.50863667980524 | 0.472509211762156 | 1.62701354721305 | 1.76893961679512 | 1.84534373934814 |
| nitrate_reduction | 0.361393852988959 | 1.10711801530257 | 1.36406329022469 | 2.27134865538856 | 2.60242003490812 | 2.92904241939844 |
| nitrogen_respiration | 0.175723616590962 | 0.50863667980524 | 0.472509211762156 | 1.64858124957876 | 1.76893961679512 | 1.84534373934814 |
| fumarate_respiration | 0 | 0 | 0 | 0 | 0 | 0 |
| intracellular_parasites | 0.265243194854282 | 0.144910734987248 | 0.481179105555957 | 0.394958549572016 | 0.41379851346316 | 0.2820907627029 |
| chlorate_reducers | 0 | 0.0405750057964294 | 0.0664691857524745 | 0.165801711936375 | 0.1078621717558 | 0.0705226906757249 |
| predatory_or_exoparasitic | 0.149199297105534 | 0.299965221423603 | 0.349685716349975 | 0.353171126238458 | 0.28828616814732 | 0.193937399358244 |
| chloroplasts | 0.0132621597427141 | 0.0101437514491073 | 0.0419044866700383 | 0.0957066792478264 | 0.10590104136024 | 0.0505412616509362 |
| cyanobacteria | 0.0265243194854282 | 0.00144910734987248 | 0.00144498229896684 | 0.0215677023657074 | 0.00196113039556 | 0.0176306726689312 |
| anoxygenic_photoautotrophy_H2_oxidizing | 0.0530486389708564 | 0.066658938094134 | 0.0231197167834694 | 0.505493024196266 | 0.55696103233904 | 0.563006147227871 |
| anoxygenic_photoautotrophy_S_oxidizing | 0 | 0.00434732204961743 | 0.0158948052886352 | 0.0444833861292714 | 0.01372791276892 | 0.0176306726689312 |
| anoxygenic_photoautotrophy_Fe_oxidizing | 0 | 0 | 0 | 0 | 0 | 0 |
| anoxygenic_photoautotrophy | 0.0530486389708564 | 0.066658938094134 | 0.0375695397731378 | 0.551324391723394 | 0.57068894510796 | 0.579461441718873 |
| oxygenic_photoautotrophy | 0.0265243194854282 | 0.00144910734987248 | 0.00144498229896684 | 0.0215677023657074 | 0.00196113039556 | 0.0176306726689312 |
| photoautotrophy | 0.0795729584562846 | 0.0681080454440065 | 0.0390145220721046 | 0.572892094089102 | 0.57265007550352 | 0.597092114387804 |
| aerobic_anoxygenic_phototrophy | 0.122674977620105 | 0.0898446556920937 | 0.0303446282783036 | 0.5324526521534 | 0.57265007550352 | 0.585338332608517 |
| photoheterotrophy | 0.603428268293492 | 0.620217945745421 | 0.618452423957807 | 1.15791602075891 | 1.06097154399796 | 1.11543389085438 |
| phototrophy | 0.62995258777892 | 0.621667053095293 | 0.619897406256773 | 1.17948372312462 | 1.06293267439352 | 1.13306456352331 |
| plastic_degradation | 0.0497330990351779 | 0.0202875028982147 | 0.0419044866700383 | 0.00539192559142684 | 0.0098056519778 | 0.00352613453378625 |
| ureolysis | 0.0928351181989987 | 0.0883955483422212 | 0.179177805071888 | 0.226460874839927 | 0.14120138848032 | 0.544200096381011 |
| reductive_acetogenesis | 0 | 0 | 0 | 0 | 0 | 0 |
| chemoheterotrophy | 41.5967640330228 | 37.9405286343612 | 35.3731666787082 | 16.4211093886904 | 15.8812339432449 | 15.3610174073508 |
| Unassigned | 9.87036238851497 | 13.3462786923255 | 16.0031789610577 | 31.2893442070499 |  |  |

Table.S3 Phenol hydroxylase gene sequences of *Acinetobacter* sp. DW-1

| Gene | Sequences |
| --- | --- |
| Phenol  hydroxylase gene | GGAGTCTATCGGAGTATGTCGGACCTGTTCGAGCAGGAACTTGATGCATTCGATGCCGAGCGTCATGTGGCGTGATTCGTCCGATTGCGCGGAGAAGCCGAACGTGACGGTCGACATGTCGCCGTTGTAGGCGGCGCCCGACATGAACGGCACGAACAGCAGGTTCGTCAGCACGTATTCGAACGAGAAGCTGACCGCGGTCAGGAACTCGAACGGCCCCGACGAATACGCATCCTCGAAGAACGACTTCGGCACCGACAGATACCAGACGCGATCGAACCAGTGGTTCGAGTGATGGAACCCGTTGAAGAACTTGTTGTAGGTCGACATCGCGTGCGTTTCGGTCTGGTAGTGCCGCAGCTCGTCGATCGACTGCATCTGGCACGCGATGCGCGCGCCCTCGCCGGTGAAGTGCCGGCCGACGTGCGCGAAGCCGCGGTGCGCGAGGTATTCGAGCGGCGTCACGCCCTGGATGAACAGCTTCAGCGCGTTGATGTAGCGGGCGTCGCTCACGCCGAGGAACGCGTTGTTCTGCGTGAACGCGTCGATCACCGCGTACAGCTTCTTTTCCTTCTCGCCCTGGTATTTCCAGTACGCATCCATCGTCAGGCGGAACGGGTCGACCCACTTGTCCCAGTCGTAT |
